# A biliary tract obstruction complicated by acute appendicitis and portal vein thrombosis-a case report and review of literature

**DOI:** 10.1016/j.ijscr.2021.106140

**Published:** 2021-06-29

**Authors:** Tomáš Řezáč, Pavel Zbořil, Katherine Vomáčková, Petr Špička

**Affiliations:** a Department of Surgery I, Faculty of Medicine and Dentistry, Palacky University Olomouc, Olomouc 77900, Czech Republic; b Department of Surgery I, University Hospital Olomouc, Olomouc 77900, Czech Republic

**Keywords:** BMI, Body Mass Index, CT, Computed Tomography, ASA score, The American Society of Anesthesiologists score, ECOG performance status, The Eastern Cooperative Oncology Group performance status, CRP, C-Reactive Protein, CR POSSUM, Colorectal Physiological and Operative Severity Score for the Enumeration of Mortality and Morbidity, ERCP, Endoscopic retrograde cholangiopancreatography, ICU, Intensive Care Unit, ALT, alanine transaminase, GGT, gamma-glutamyl transpeptidase, Acute appendicitis, Pylephlebitis, Portal vein thrombosis, Treatment, Case report

## Abstract

**Introduction and importance:**

Acute appendicitis is one of the most common surgical diagnoses in clinical practice. In case of uncomplicated course, diagnosis and treatment do not cause significant difficulties. On the other hand, unrecognized or complicated appendicitis can rarely bring unusual complications that threaten the patient with delayed treatment rather than the course itself. Portal vein thrombosis, also known as pylephlebitis, with an incidence of 1/1000 acute admissions, certainly meets this statement.

**Case presentation:**

In this study, we present a successful treatment of advanced acute appendicitis complicating treatment of biliary obstruction. Due to the advanced inflammation with forced intestinal resection in the extent of right-sided hemicolectomy. And then successful conservative treatment of portal vein thrombosis in the surgical facility lasting a total of 6 weeks when the patient was discharged to home care without sequelae.

**Clinical discussion:**

The epidemiology, presentation, diagnosis and strategy of treatments as well as their outcomes were discussed.

**Conclusion:**

Portal vein thrombosis after acute appendicitis is rare. In case of unfavorable postoperative course with high inflammatory markers, temperatures, discomfort and abdominal pain, a CT scan is in order, which can easily establish the diagnosis and subsequently target the treatment in the right direction. Treatment of pylephlebitis is conservative and long term. It consists in the application of low molecular weight heparin and targeted antibiotic treatment. The mortality rate is 32%.

## Highlights

• Appendicitis following cholangoitis
• Right hemicolectomy
• Pylephlebitis
• Treatment

## 1 Introduction and importance

Common complications of acute appendicitis as well as its diagnosis and therapy do not cause us any difficulties. However, rare complications are described in the literature, occurring in units of cases and at different intervals from the procedure, which may lead to diagnostic confusion, as well as their treatment may not be standard. Vascular complications involving both intestinal necrosis and venous thrombosis have been described. Inflammatory complications range from inflammation of the abdominal wall to variously localized abscesses. And finally, perhaps not so rare, stump appendicitis. Advanced appendicitis causing intestinal necrosis on account of small vein thrombosis may not in itself impose a significant burden on the patient, perhaps only complications arising from intestinal resection. However, the ascending thrombosis of the portal vein, either due to appendicitis or cholangitis, is already a certain complication, moreover with significant morbidity. Despite treatment, it is fatal in up to 32% of cases and has a significantly worse prognosis if left untreated. Only a few cases of predominantly pylephlebitis following acute appendicitis have been described in the available literature. In this report, we describe a case of a patient with concomitant cholangitis, appendicitis, and portal vein thrombosis. This paper has been reported in line with the SCARE 2020 criteria [1].

## 2 Case presentation

We present the case of a 49-year-old male patient, a non-smoker with a BMI of 33, a history of knee arthroscopy, and treated for hypothyroidism. No family burden was noted. The patient was seen in the emergency department of a university hospital for right subcostal pain spreading to the back and epigastrium after a dietary mistake. Among other complaints, he reported a temperature with a maximum of 37.5st C.

**Fig. 1:**
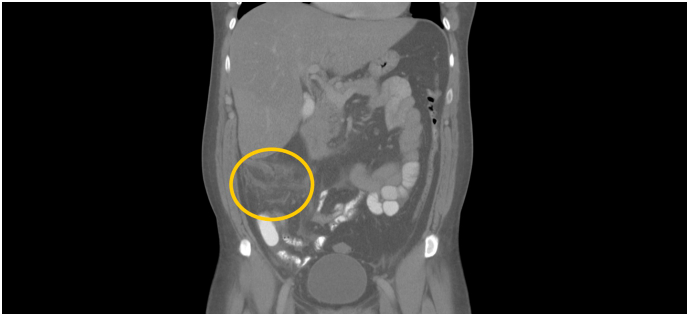
CT scan of acute subhepatal appendicitis with abscess.

**Fig. 2:**
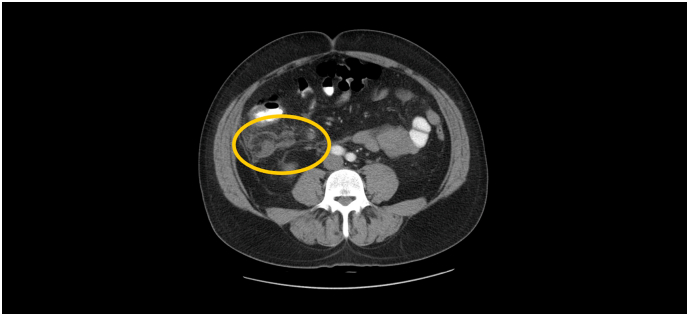
CT scan of acute subhepatal appendicitis with abscess.

Laboratory showed only mild inflammation (CRP 15.6 mG/L, white blood cell count 13.7 × 106/L). Ultrasound and subsequent CT scan confirmed bile duct dilatation with no evidence of other pathology in the abdominal cavity. The patient was admitted to the gastroenterology department and underwent ERCP examination with papillosphincterotomy and biliary sludge extraction. The procedure was performed by an expert endoscopist with 20 years of experience and well-tolerated by the patient. In the following course, despite antibiotic therapy, the clinical condition deteriorated. Therefore, a follow-up CT scan was performed four days after the procedure and described advanced appendicitis with subhepatically located appendix. Fig. 1, Fig. 2. The patient was referred for acute revision and transferred to the surgical ward. The procedure was performed the same day by the head of the department. Due to the advanced findings, perioperative necrosis of the colon ascendens with mesocolic vein thrombosis was found, at the cost of open right-sided hemicolectomy, and the patient was transferred to the intensive care unit.

**Fig. 3:**
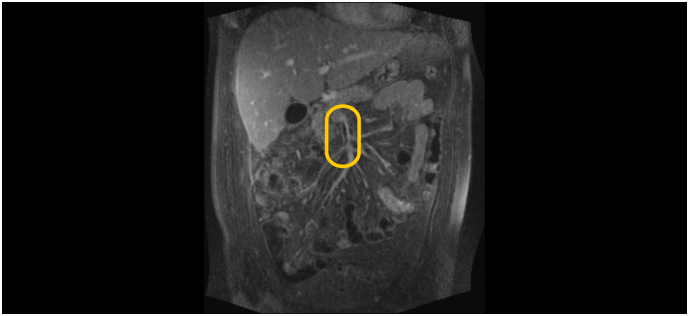
MRI scan of portal vein thrombosis.

Targeted antibiotic therapy was administered, *Proteus mirabilis* was repeatedly cultivated. However, the condition was still not favorable, a follow-up postoperative CT scan 5 days apart revealed an intra-abdominal abscess which was drained under CT control, and portal vein thrombosis which was treated conservatively. Fig. 3. Both Clavien Dindo grade IIIa. The patient was treated with imipenem/cilastatin 500 mg/500 mg antibiotics 4 times daily for 14 days and subcutaneous application of low molecular weight nadroparin at the therapeutic dose (Fraxiparine©). Further course in the standard ward was already without complications. The drain was flushed regularly and withdrawn when there was no secretion. The patient was discharged in good clinical condition to home care provided with oral antibiotics sultamicillin 375 mg 2 times daily for 1 month and nadroparin for 3 months. The entire hospitalization lasted 56 days. At follow-up, the patient was free of problems, and a follow-up ambulance colonoscopy was performed 5 years later and showed no signs of pathology. The patient developed a incisional hernia about one year after surgery, but it does not cause any problems and so far he prefers conservative management.

## 3 Clinical discussion

In general, complications can be divided into early and late. Leaving aside the common ones such as an abscess or surgical wound infection, thrombosis of the ileocolic or portal vein associated with intestinal necrosis is rarely observed. Pylephlebitis or portal vein thrombosis is a rare intra-abdominal vascular complication. The incidence is reported to be 1/1000 acute admissions [2] where the predominant symptom is abdominal pain. It arises as phlebitis of the small veins draining the infectious focus and progresses with inflammation to the development of portal vein thrombophlebitis. Up to 70% is caused by acute diverticulitis [3]. Pylephlebitis was more frequently described in the early 20th century due to no or only minimal antibiotic therapy options. Today, thanks to significant advances in this field, the incidence is virtually zero. The diagnosis is quite complicated due to mild and non-specific symptoms. Abdominal pain, temperature, leukocytosis, and elevation of liver enzymes, especially ALT and GGT, are most common; nausea and vomiting are rare. Imaging examinations are predominated by contrast-enhanced CT scan which may be supplemented by MRI but without further diagnostic benefit. The thrombus is usually only partially obstructing the lumen, slows the venous blood flow, and maybe a source of liver abscesses in case of bacterial contamination (88% of cases) [3]. Here, diagnosis usually does not cause difficulties, treatment is drainage in collaboration with an interventional radiologist and with the help of antibiotics. Multilocular abscesses can be a challenge. The most commonly cultured species are the Bacteroides and Escherichia. Of attraction is that the Bacteroides species contains heparin-degrading enzymes and activates fibrin formation, thus has a procoagulant effect. Less common are *Proteus mirabilis*, *Klebsiella pneumoniae*, anaerobic streptococci, and clostridia [4]. Up to 42% of cases present with intestinal ischemia to necrosis [5]. It manifests in the early postoperative period with clinical deterioration, pain, and melena. The treatment is surgical, resection of the affected intestinal section according to the drainage area. Surgical thrombectomy is not recommended, although it can rapidly recanalize the lumen, this is mainly due to damage to the vascular wall and the risk of retrombosis. [6]. CT scan with intravenously administered contrast agent is the primary imaging modality. Here again, early diagnosis is the prevention of major complications.

Treatment of pylephlebitis is mainly antibiotics and anticoagulation. Primarily empirical curative dose of antibiotics, which is subsequently adjusted according to the cultivation result, as well as a therapeutic dose of low molecular weight heparin as early as the postoperative condition allows. Treatment is long-term, antibiotic 4–6 weeks, anticoagulant 3–6 months [7]. The mortality rate of the disease is up to 32% even today [5], [8].

## 4 Conclusion

Acute cholangitis or appendicitis does not seem to be a major complication of routine medical practice, this statement is especially true for an uncomplicated course. However, problems, both diagnostic and therapeutic, may arise in the case of an atypical course or coexisting disease, as well as in the presence of rare complications. The insidiousness, incidence, and mortality of pyelophlebitis are certainly worth mentioning. It is also worthy of our attention, especially in identifying the cause of atypical postoperative course.

## Provenance and peer review

Not commissioned, externally peer-reviewed.

## Sources of funding

Supported by 10.13039/501100003243Ministry of Health, Czech Republic – conceptual development of research organization (FNOl, 00098892).

## Ethical approval

Not required in our institution to publish anonymous case reports.

## Consent

Written informed consent was obtained from the patient for publication of this case report and accompanying images. A copy of the written consent is available for review by the Editor-in-Chief of this journal on request.

## Research registration

Not applicable.

## Guarantor

MUDr Tomáš Řezáč Ph.D.

## CRediT authorship contribution statement

Tomáš Řezáč: design of the study, collection of the data, drafting the manuscript, final approval of the version to be submitted.

Pavel Zbořil: revising the manuscript, final approval of the version to be submitted.

Katherine Vomáčková: revising the manuscript, english correction, final approval of the version to be submitted.

Petr Špička: revising the manuscript, final approval of the version to be submitted.

## Declaration of competing interest

The authors declare that they have no conflict of interest related to individual authors' commitments.

The authors declare no potential conflicts of interest related to commitments of editors, journal staff, or reviewers.
